# Current state of open access to journal publications from the University of Zagreb School of Medicine

**DOI:** 10.3325/cmj.2016.57.71

**Published:** 2016-02

**Authors:** Lea Škorić, Dina Vrkić, Jelka Petrak

**Affiliations:** Central Medical Library, University of Zagreb School of Medicine, Zagreb, Croatia

## Abstract

**Aims:**

To identify the share of open access (OA) papers in the total number of journal publications authored by the members of the University of Zagreb School of Medicine (UZSM) in 2014.

**Methods:**

Bibliographic data on 543 UZSM papers published in 2014 were collected using PubMed advanced search strategies and manual data collection methods. The items that had “free full text” icons were considered as gold OA papers. Their OA availability was checked using the provided link to full-text. The rest of the UZSM papers were analyzed for potential green OA through self-archiving in institutional repository. Papers published by Croatian journals were particularly analyzed.

**Results:**

Full texts of approximately 65% of all UZSM papers were freely available. Most of them were published in gold OA journals (55% of all UZSM papers or 85% of all UZSM OA papers). In the UZSM repository, there were additional 52 freely available authors’ manuscripts from subscription-based journals (10% of all UZSM papers or 15% of all UZSM OA papers).

**Conclusion:**

The overall proportion of OA in our study is higher than in similar studies, but only half of gold OA papers are accessible via PubMed directly. The results of our study indicate that increased quality of metadata and linking of the bibliographic records to full texts could assure better visibility. Moreover, only a quarter of papers from subscription-based journals that allow self-archiving are deposited in the UZSM repository. We believe that UZSM should consider mandating all faculty members to deposit their publications in UZSM OA repository to increase visibility and improve access to its scientific output.

Open access (OA) availability and searchability of new research results in medicine have a significant and positive impact, from research and education to everyday medical practice (1). In the last decades, numerous efforts have been made to promote OA as a reliable and efficient model for communication, dissemination, and deposit of medical information. The most noteworthy examples are Budapest Initiative (2) and Bethesda Statement (3), as well as OA mandates of prominent medical institutions and organizations (4,5), such as PubMed (6) and PubMed Central (7), Biomed Central (8), and PLoS journals (9). There is evidence on a tremendous growth of OA in the last years. For example, Laakso and Björk (10) showed that during 2011, papers published in biomedical journals constituted 35.5% of the total number of OA papers. Simple browsing of the Directory of Open Access Journals (DOAJ) in January 2016 has confirmed that OA is rooted in biomedicine more deeply than in any other science and technology discipline, with 2006 OA journals (18% of all journals included) and 670 883 OA papers (31% of all papers included) (11). OA is usually categorized into gold OA (fully and freely accessible papers in OA journals), green OA (authors’ self-archiving of submitted and accepted versions of their manuscripts), and hybrid OA (individual papers made OA in journals that are otherwise subscription-only) (12).

Gargouri et al (13) found that the share of green OA papers among all papers in biomedical research indexed in Thomson-Reuters databases from 2005 to 2010 was 6%, in clinical medicine 9%, and in health 12%, while the share of gold OA was 8% in biomedical research and 5% in both clinical medicine and health. Archambault et al (14) found that the share of green and hybrid OA papers in biomedical research, clinical medicine, and public health included in DOAJ, PubMed Central, and Scopus from 2008 to 2011 was 51%, 36%, and 35% respectively, and that the share of gold OA was 11% in both, biomedical research and clinical medicine, and 13% in the public health.

Support and practice of OA has been growing in Croatia as well. The UNESCO’s Global Open Access Portal (GOAP) (15) mentions Croatia as an active OA publishing community with a national open-access journal platform Hrčak (16), seven institutional OA repositories, national Declaration on Open Access published in 2012 (17), but just one institutional mandate (18). Moreover, GOAP named the University of Zagreb School of Medicine (UZSM) one of the key organizations in the Croatian OA environment. UZMS is the Croatian largest educational and research medical institution, and characteristics of its publication output may be considered representative of the national medical academic community. The *Croatian Medical Journal*, partly owned by the UZSM, has been using diamond open access model since 1997 (19) and has been included in PubMed Central since 2006. On the other hand, the UZSM employs green OA through its institutional repository, established in 2006 (20).

The primary aims of this study were to identify the proportion of OA papers in the total number of journal publications authored by faculty members of the UZSM and indexed by PubMed in 2014 and determine the type of OA they belong to. This was done in order to examine if UZSM follows global trends in medical OA publishing and to identify the potential differences. Since all 12 Croatian journals indexed by PubMed comply with the national policy recommending OA to the results of publicly funded research, an additional aim was to analyze their availability and visibility in a broader international context.

## Methods

We searched PubMed, a freely available biomedical bibliographic database that regularly covers more than 5500 medical journals. Our data set included papers from PubMed published between January 1 and December 31, 2014, in which at least one author was affiliated to UZSM. PubMed was searched by combination of the most frequently used variations of affiliation name (Zagreb AND (school of medicine OR medical school OR medical faculty OR medicinski fakultet OR university hospital)) and publication date (2014). We double-checked the author names in each paper retrieved using the official UZSM teaching staff list.

Close examination of search results revealed the most important methodological limitation of our research. It showed that many papers published in a number of PubMed-indexed Croatian journals were not retrieved. The main reason was that the authors’ affiliation data in these records were incomplete. We browsed all PubMed records from Croatian journals published in 2014 and manually added papers authored by UZSM teaching staff to the initial search results. Since there could be more records with incomplete affiliation data in PubMed, we can only assume that most PubMed papers published by the UZSM in other indexed journals were included in our study.

The final set of UZSM affiliated papers was searched again using the PubMed filter “free full-text.” Each retrieved record that had “free full text” or PubMed Central (PMC) icons was considered as an OA paper. We checked the provided link to determine whether the full text was freely available.

In the second stage of our study, we investigated the rest of the UZSM papers. In order to find out how many of them are potentially available via green OA, we investigated copyright and self-archiving policies of the respective journals. We used journals’ web pages and Sherpa-Romeo, a searchable database of publishers ' policies regarding self-archiving of journal papers on the web and in OA repositories (21). We found that a large number of papers without PubMed “free full text” link were freely available through journals’ web pages or other official web platforms (excluding institutional repositories, blogs, or social networks). Among them there were also papers published in a number of Croatian biomedical journals. Since there was no link from their PubMed record to the full text source, they were not selected by the “free full text” filter. This confirms the importance of using manual data collection in OA studies because data available from indexes only provide part of the total picture (10).

## Results

The combination of PubMed search and browsing methods resulted in 543 records of papers published in 2014 with at least one author affiliated to the UZSM. Two thirds of these papers (360 or 66.3%) were published in foreign medical journals and one third (183 or 33.7%) in 10 of 12 Croatian journals (in two journals there were no papers authored by UZSM faculty).

The repeated search using the “free full-text” filter found 149 OA papers (27.4% of all papers retrieved). Out of them, 38 papers (25.5%) were published by Croatian OA biomedical journals (*Croatian Medical Journal*, *Biochemia Medica,* and *Psychiatria Danubina*). The rest were published by international OA journals: 88 papers in gold OA journals (59.1%) and 23 papers (15.4%) in hybrid journals, mainly by prominent publishers, like Elsevier, Oxford University Press, Springer. etc. All of these papers had a direct PubMed link to their full-text version either in the PubMed Central archive or on the journals’ web pages.

Since we were aware that almost all Croatian scientific journals used gold OA publishing model, we checked the actual OA availability of the rest of the papers from Croatian journals in the UZSM 2014 set. At the Hrčak platform we found freely accessible full-text versions of additional 108 PubMed-indexed papers. The only indexed journal from our Croatian set that did not archive papers in Hrčak database was *Liječnički vjesnik*, but free full-texts of all 37 PubMed-indexed UZSM papers published in this journal were accessible at the journal's web page. This showed that all Croatian journals indexed in PubMed used gold OA, but only 20.8% of papers had a direct link from PubMed.

In the second stage of our research, while investigating journal publishers copyright and self-archiving policies, 6 more freely available papers, published by foreign journals, were found. With all these manually retrieved records of OA papers added to the PubMed “free-full text” results, the total share of gold open access papers increased to 55.2%.

The subscription-based journals published 243 UZSM papers (44.8%). According to Sherpa-Romeo and journal publishers’ web pages, authors of 196 papers were allowed to archive the accepted manuscript of their papers (the so called post-print or final draft post-refereeing) on their own websites and/or institutional repositories. This represents 80.7% of all UZSM papers published in subscription-based journals. Many of the papers available for green OA in our study were published by major commercial publishers like Elsevier or Springer, and most of them allow public release of full texts after an embargo period. The embargo period for scientific, technical, and medical fields varies from 6 to 36 months, but it is most often 12 months. At the time we started this research, there were 52 authors’ manuscripts from subscription-based journals archived and freely accessible in the UZSM institutional repository.

**Figure 1:**
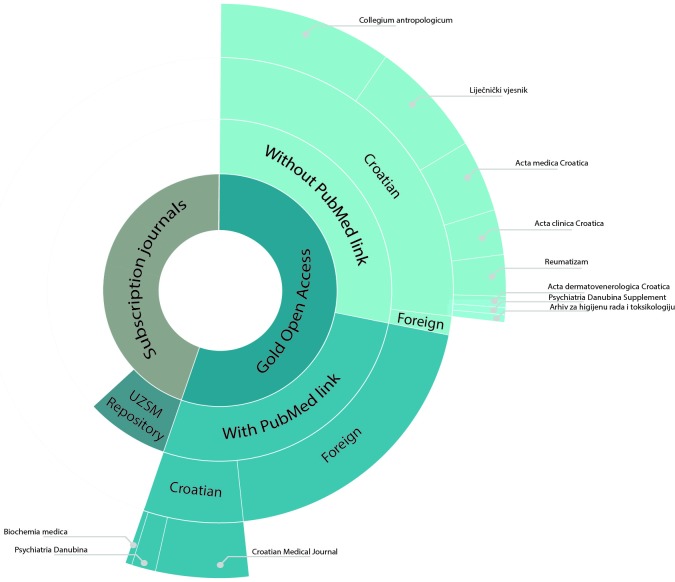
Distribution of PubMed papers authored by members of UZSM in 2014 according to their OA availability, methods of achieving OA and journal origin.

Altogether, 352 OA UZMS papers (64.8%) published in 2014 had full texts available across different types of outlets (Figure 1). Keeping in mind that some of the co-authors from other institutions could have posted some of these papers in their institutional repositories, the share of available full texts could be even bigger.

## Discussion

The overall proportion of OA papers in our study was 64.8%. It is higher than in a study examining OA availability in the members of European Research Area and four additional countries (14). In the period 2008-2011, the proportion of OA papers at the world level was 43%, and the Croatian sample with 60% of OA papers was the third largest (following Macedonia and Brazil).

Also, the share of gold OA in our study (55.2%) is greater than in a similar study analyzing real and potential OA papers produced by university medical departments, where gold OA proportion varied between 27.8 and 42.1% (22).

However, when we compare our results with the share of records with a “free full text” link among all PubMed indexed papers published in 2014, the situation is not so satisfactory. The percentage of UZSM papers with a “free full text” link was 27.4%, and the respective share for all PubMed-indexed papers was 37.6%. In fact, only half of UZSM gold OA papers are accessible directly via PubMed. Moreover, although papers published by Croatian journals were 100% OA, only 20.8% had the PubMed “free full text” link.

Considering that more than half of the analyzed papers (59.3%) are written in English, it is against the interests of both authors and journals that international PubMed users’ community has no indication of their free and open accessibility. Moreover, it is also unfavorable for the visibility and impact of papers written in the Croatian language, because open access can reduce language barriers by eliminating restrictions on re-publication and re-use (including translations) (23).

One of the reasons why nearly all Croatian scientific journals are open access may be found in their funding model. They are all supported by the governmental funds following criteria set for each scientific domain (24). In the field of biomedicine, the most important criteria are indexing by the international bibliographic databases (eg, Medline is highly rated) and SCImago quartile values. Free online access on Hrčak or on journals’ web pages is also listed and taken into account. The results of our study indicate that better journals’ visibility could be achieved by more active approach to indexing database producers, increased quality of metadata, and linking of the bibliographic records to full texts.

However, is such a high proportion of OA a reflection of authors’ choice? With the exception of those UZSM authors who are recipients of the international research grants subject to a mandate of OA publishing or archiving, we believe that our authors do not select journals for publishing their papers according to journals’ access model. Though OA to journal papers increases their visibility and impact (25,26), authors predominantly base their selection on journals’ indexing status and metric indicators, complying with the official national criteria for academic advancement.

Finally, could the rate of OA at UZSM be even higher? Some of UZSM authors participate in contemporary information sharing practices of academic and research community by posting full texts of their papers in institutional repository (green OA) or on social networking sites. However, most of our authors are still not aware of all OA benefits. This research showed that only 26.5% full texts of papers from subscription-based journals that allow open access are deposited in the UZSM repository. This corresponds to the results of Gargouri et al (27), who found that only about 15%-20% of papers were spontaneously self-archived compared to 60% archived by institutions with OA mandate. Therefore, based on the recommendations of the European Commission (28,29) and national strategic documents (17), UZSM and other Croatian medical schools should consider implementation of OA mandate policies for their teaching and research staff as an efficient method for achieving better visibility of their scientific output (30).

## References

[1] Hojgaard L, Omling P. Open access in biomedical research. Strasbourg: European Science Foundation; 2012. p. 24. (Science Policy Briefing; No. 47). Available from: http://www.esf.org/fileadmin*/Public_documents/Publications/spb47_OpenAccess.pdf*. Accessed: January 15, 2016.

[2] Budapest Open Access Initiative. Budapest; 2002 Feb 14. Available from: http://www.budapestopenaccessinitiative.org/read*.* Accessed: January 25, 2016.

[3] Suber P, Brown PO, Cabell D, Chakravarti A, Cohen B, Delamothe T, et al. Bethesda Statement on Open Access Publishing. 2003 Jun 20. Available from: http://legacy.earlham.edu/~peters/fos/bethesda.htm*.* Accessed: January 25, 2016.

[4] U.S. Department of Health & Human services. NIH Public Access Policy. Available from: https://publicaccess.nih.gov/*.* Accessed: January 25, 2016.

[5] Trust W. Open Access Policy: Position statement in support of open access to published research. Available from: http://www.wellcome.ac.uk/About-us/Policy/Spotlight-issues/Open-access/Policy/index.htm*.* Accessed: January 8, 2016.

[6] PubMed. Bethesda (MD): U.S. National Library of Medicine. Available from: http://www.ncbi.nlm.nih.gov/pubmed*.* Accessed: December 5, 2015.

[7] PMC. PMC Overview. Bethesda (MD): U.S. National Library of Medicine. Available from: http://www.ncbi.nlm.nih.gov/pmc/about/intro/. Accessed: December 5, 2015.

[8] BioMed Central. About BioMed Central; 2015. Available from: https://www.biomedcentral.com/about*.* Accessed: December 5, 2015.

[9] PLOS. PLOS Open Access. Available from: https://www.plos.org/open-access/*.* Accessed: January 25, 2016.

[10] Laakso M, Björk BC (2012). Anatomy of open access publishing: a study of longitudinal development and internal structure.. BMC Med.

[11] DOAJ. Directory of Open Access Journals. Lund: Lund University; c2016. Available from: https://doaj.org/*.* Accessed: January 24, 2016.

[12] Chatterjee P, Biswas T, Mishra V (2013). Open access: the changing face of scientific publishing.. J Family Med Prim Care..

[13] Gargouri Y, Lariviere V, Gingras Y, Carr L, Harnad S. Green and gold open access percentages and growth, by discipline. Available from: http://arxiv.org/abs/1206.3664*.* Accessed: December 6, 2015.

[14] Archambault E, Amyot D, Deschamps P, Nicol A, Rebout L, Roberge G. Proportion of open access peer-reviewed papers at the European and World levels — 2004-2011. Brussels; Montreal; Washington: Science Matrix Inc. 2103, pp 14. Available from: http://www.science-metrix.com/pdf/SM_EC_OA_Availability_2004-2011.pdf*.* Accessed: January 24, 2016.

[15] Unesco. Global Open Access Portal. Paris: UNESCO; c2015. Available from: http://www.unesco.org/new/en/communication-and-information/portals-and-platforms/goap/access-by-region/europe-and-north-america/croatia/*.* Accessed: December 13, 2015.

[16] Stojanovski J, Petrak J, Macan B (2009). The Croatian national open access journal platform.. Learn Publ.

[17] Budin L, Silobrčić V, Flego G, Grgić M, Šimić D, Stojanovski J, et al. Croatian Declaration on Open Access. 2012. Available from:http://www.fer.unizg.hr/oa2012/declaration*.* Accessed: December 8, 2015.

[18] Stojanovski J. Ruđer Bošković Institute adopts open access mandate (Croatia). OpenAIRE Blog. Available from:https://blogs.openaire.eu/?p=177*.* Accessed: December 15, 2015.

[19] Barić H, Polšek D, Andijašević L, Gajović S (2013). Open access – is this the future of medical publishing?. Croat Med J.

[20] Markulin H, Šember M (2014). University of Zagreb Medical School Repository: promoting institutional visibility.. Croat Med J.

[21] University of Nottingham. Sherpa/Romeo. Nottingham: University of Nottingham; c2006-2015. Available from: http://www.sherpa.ac.uk/romeo/index.php?la=en&fIDnum=|&mode=simple*.* Accessed: December 8, 2015.

[22] Bongiovani P, Miguel S, Gómez ND. Open Access, scientific impact and the scientific production in two Argentine universities in the field of medicine. Revista Cubana de Informacion en Sciencias de la Salud. 2013 Feb [cited 2016 Feb 3];24(2). Available from: http://www.acimed.sld.cu/index.php/acimed/article/view/428/300*.* Accessed: February 3, 2016.

[23] Glasziou P (2014). The role of open access in reducing waste in medical research.. PLoS Med.

[24] Ministry of Science Education and Sports of the Republic of Croatia. Criteria for MSES financial support to scientific journals [in Croatian]. c2004-20013. Available from: http://public.mzos.hr/Default.aspx?sec=2142*.* Accessed: January 15, 2016.

[25] Hebrang Grgić I (2014). Scholarly journals at the periphery: the case of Croatia.. Learn Publ.

[26] Davis PM, Walters WH (2011). The impact of free access to the scientific literature: a review of recent research.. J Med Libr Assoc.

[27] Gargouri Y, Hajjem C, Lariviere V, Gingras Y, Carr L, Brody T (2010). Self-selected or mandated, open access increases citation impact for higher quality research.. PLoS ONE.

[28] European Commission Directorate General for Research and Innovation. Guidelines on open access to scientific publications and research data in horizon 2020. Version 2.1. 2016, Feb. 15. Available from: https://ec.europa.eu/research/participants/data/ref/h2020/grants_manual/hi/oa_pilot/h2020-hi-oa-pilot-guide_en.pdf. Accessed: February 19, 2016.

[29] European Commission. Commission recommendation of 17.7.2012 on access to and preservation of scientific information. Brussels, 17.7.2012 c(2012) 4890 final. Available from: http://ec.europa.eu/research/science-society/document_library/pdf_06/recommendation-access-and-preservation-scientific-information_en.pdf*.* Accessed: February 18, 2016.

[30] Xia J, Gilchrist SB, Smith NXP, Kingery JA, Radecki JR, Wilhelm ML, et al. A review of open access self-archiving mandate policies. Portal: Libraries and Academy. The Johns Hopkins University Press; 2012;12(1):85–102. Available from: https://muse.jhu.edu/journals/portal_libraries_and_the_academy/v012/12.1.xia.html*.* Accessed: January 25, 2016.

